# Correction: Podlutskii et al. *Arabidopsis thaliana* Accessions from the Chernobyl Exclusion Zone Show Decreased Sensitivity to Additional Acute Irradiation. *Plants* 2022, *11*, 3142

**DOI:** 10.3390/plants13070947

**Published:** 2024-03-25

**Authors:** Mikhail Podlutskii, Darya Babina, Marina Podobed, Ekaterina Bondarenko, Sofia Bitarishvili, Yana Blinova, Ekaterina Shesterikova, Alexander Prazyan, Larisa Turchin, Dmitrii Garbaruk, Maxim Kudin, Gustavo T. Duarte, Polina Volkova

**Affiliations:** 1 Russian Institute of Radiology and Agroecology, 249032 Obninsk, Russia; 2 Polesye State Radiation-Ecological Reserve, 247618 Khoiniki, Belarus; 3 Belgian Nuclear Research Centre (SCK CEN), Unit for Biosphere Impact Studies, 2400 Mol, Belgium; 4 Independent Researcher, 2440 Geel, Belgium

In the original publication [1], there was a mistake in “Figure 1. Map of the experimental plots (Babchin, Vygrebnaya Sloboda, and Masany) located in the Polesye State Radiation-Ecological Reserve (Khoiniki, Gomel Region, Republic of Belarus) where A. thaliana Bab-0, VS-0, and Masa-0 natural accessions, respectively, were collected” as published.

Instead of μSv × h^−1^, mSv × h^−1^ was introduced. The corrected Figure 1 appears below.

**Figure 1:**
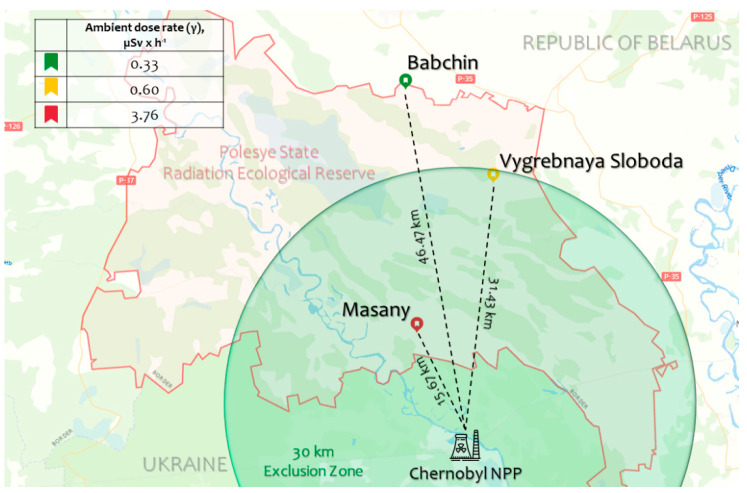
Map of the experimental plots (Babchin, Vygrebnaya Sloboda, and Masany) located in the Polesye State Radiation-Ecological Reserve (Khoiniki, Gomel Region, Republic of Belarus) where *A. thaliana* Bab-0, VS-0, and Masa-0 natural accessions, respectively, were collected. The dose rates are represented in Table 1. The map was created using Google Maps (Google LLC, Mountain View, CA, USA), and adapted with Microsoft PowerPoint 2019 (Microsoft Corporation, Albuquerque, NM, USA).

There was an error in the original publication. Due to a technical error during translation, instead of microsievert (μSv), in two places in the manuscript, we had millisievert (mSv), a 1000-times-higher dose. A correction has been made to the subsection “**2. Results**, *2.1. Arabidopsis Natural Accessions in the Chernobyl Exclusion Zone*, Header of Table 2”, from “mSv × h^−1^” to “μSv × h^−1^”. A correction has been made to the subsection “**5. Materials and Methods**, *5.1. Sampling in the Chernobyl Exclusion Zone*”, to Paragraph 2, from “mSv × h^−1^” to “μSv × h^−1^”.

The authors state that the scientific conclusions are unaffected. This correction was approved by the Academic Editor. The original publication has also been updated.
