# Supplementary material for: Arabidopsis thaliana Accessions from the Chernobyl Exclusion Zone Show Decreased Sensitivity to Additional Acute Irradiation
Source: Plants (Basel). 2022 Nov 17;11(22):3142. doi: 10.3390/plants11223142 (PMC9697804; doi:10.3390/plants11223142)

Figure S1. The enriched GO terms for downregulated genes in the chronically irradiated *A. thaliana* accession VS-0 in comparison with the reference accession Bab-0 (Biological Process dictionary)

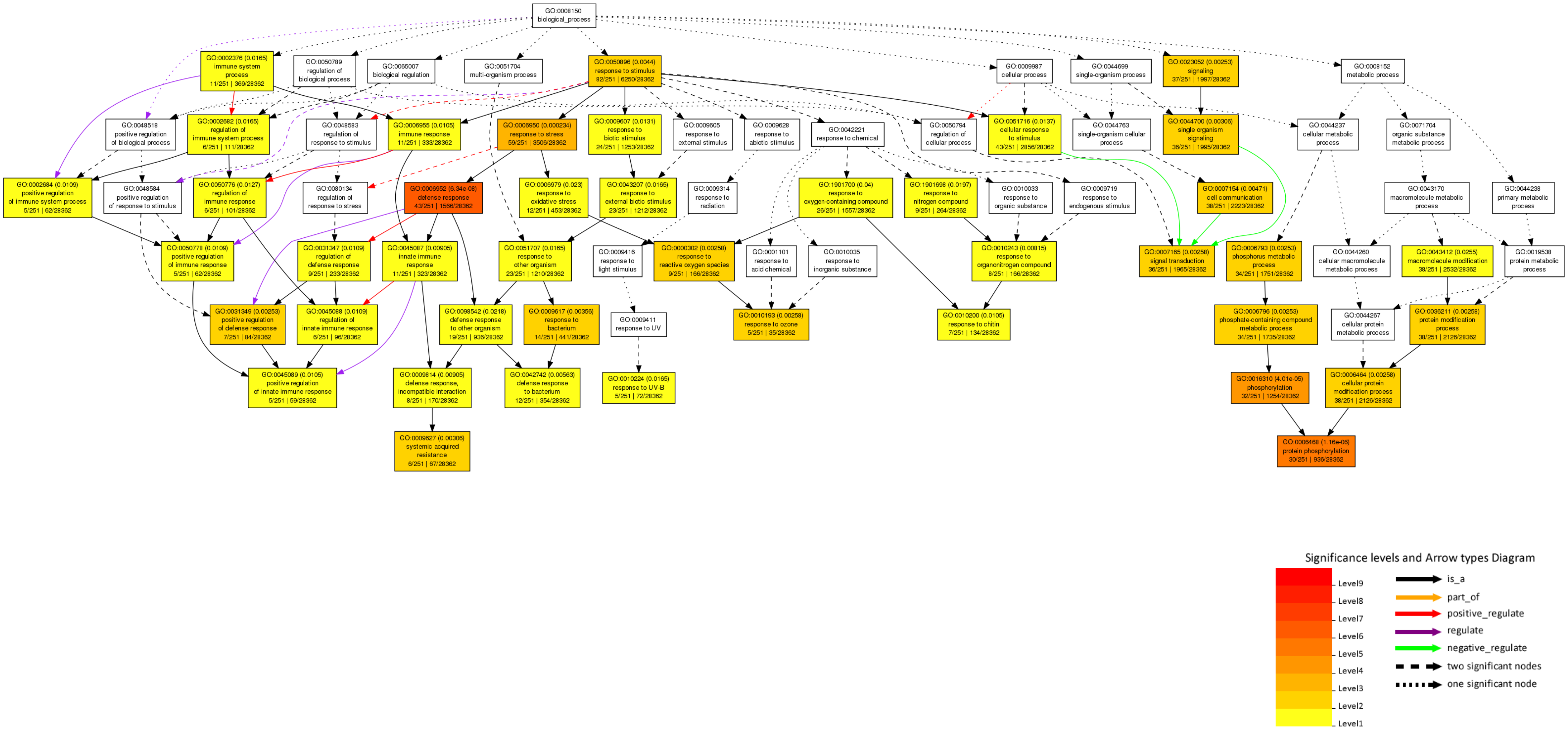

Figure S2. The enriched GO terms for downregulated genes in the chronically irradiated *A. thaliana* accession VS-0 in comparison with the reference accession Bab-0 (Molecular Function dictionary)

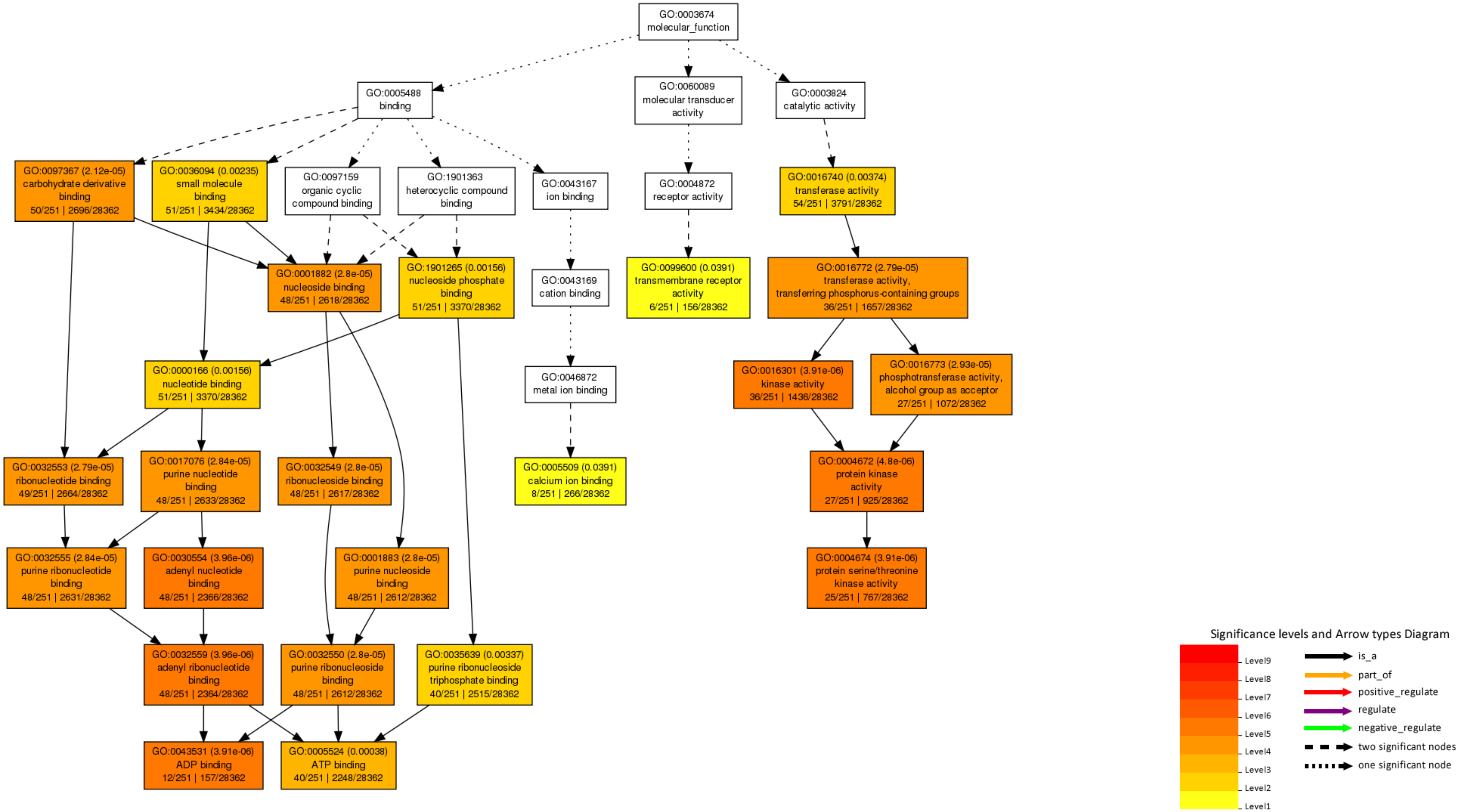

Figure S3. The enriched GO terms for upregulated genes in the chronically irradiated *A. thaliana* accession Masa-0 in comparison with the reference accession Bab-0 (Biological Process dictionary)

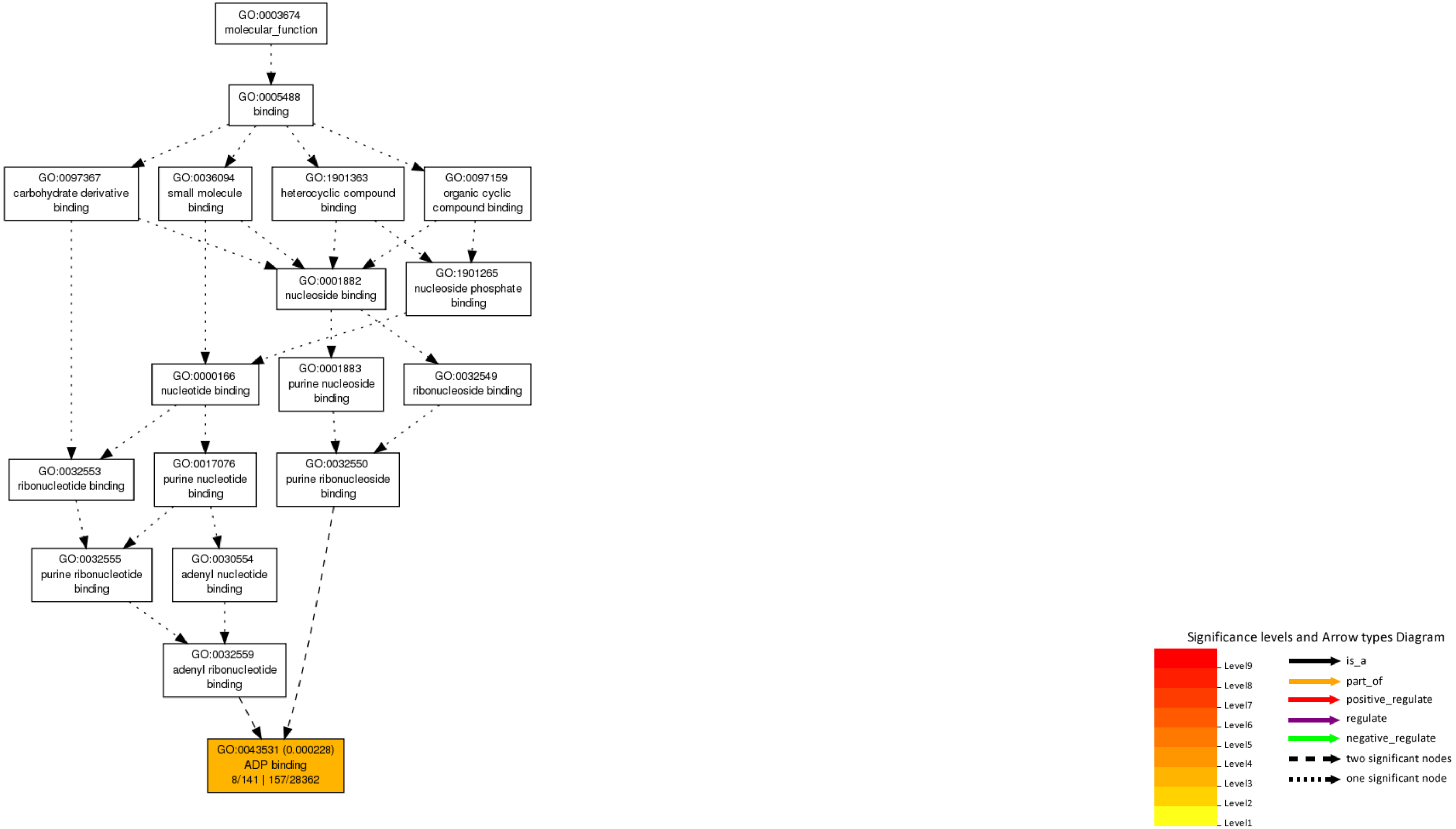

Figure S4. The enriched GO terms for downregulated genes in the chronically irradiated *A. thaliana* accession Masa-0 in comparison with the reference accession Bab-0 (Molecular Function dictionary)

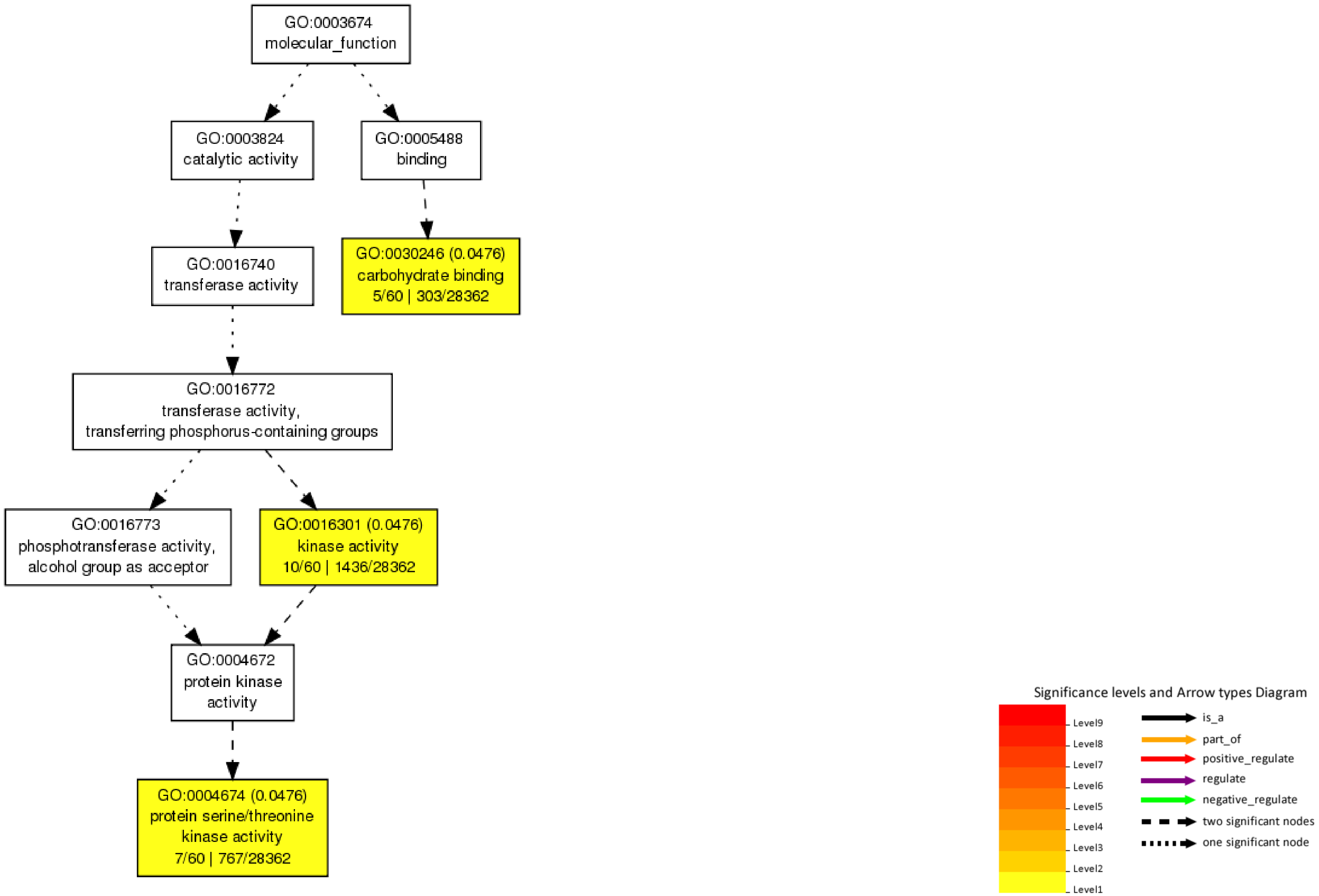

Figure S5. The enriched GO terms for upregulated genes after additional γ-irradiation of seeds from the accession Bab-0 in comparison with the reference non-irradiated seeds of the same accession (Biological Process dictionary)

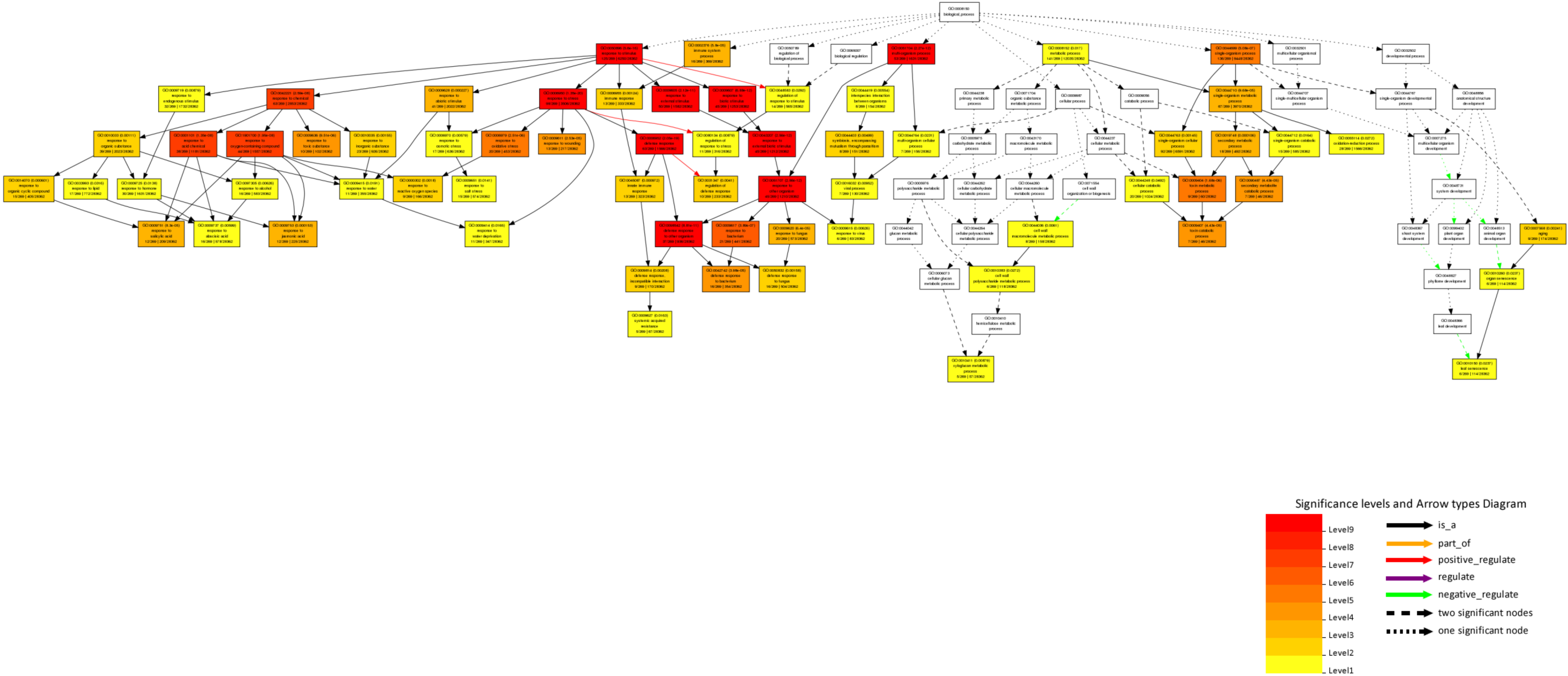

Figure S6. The enriched GO terms for downregulated genes after additional γ-irradiation of seeds from the accession Bab-0 in comparison with the reference non-irradiated seeds of the same accession (Biological Process dictionary)

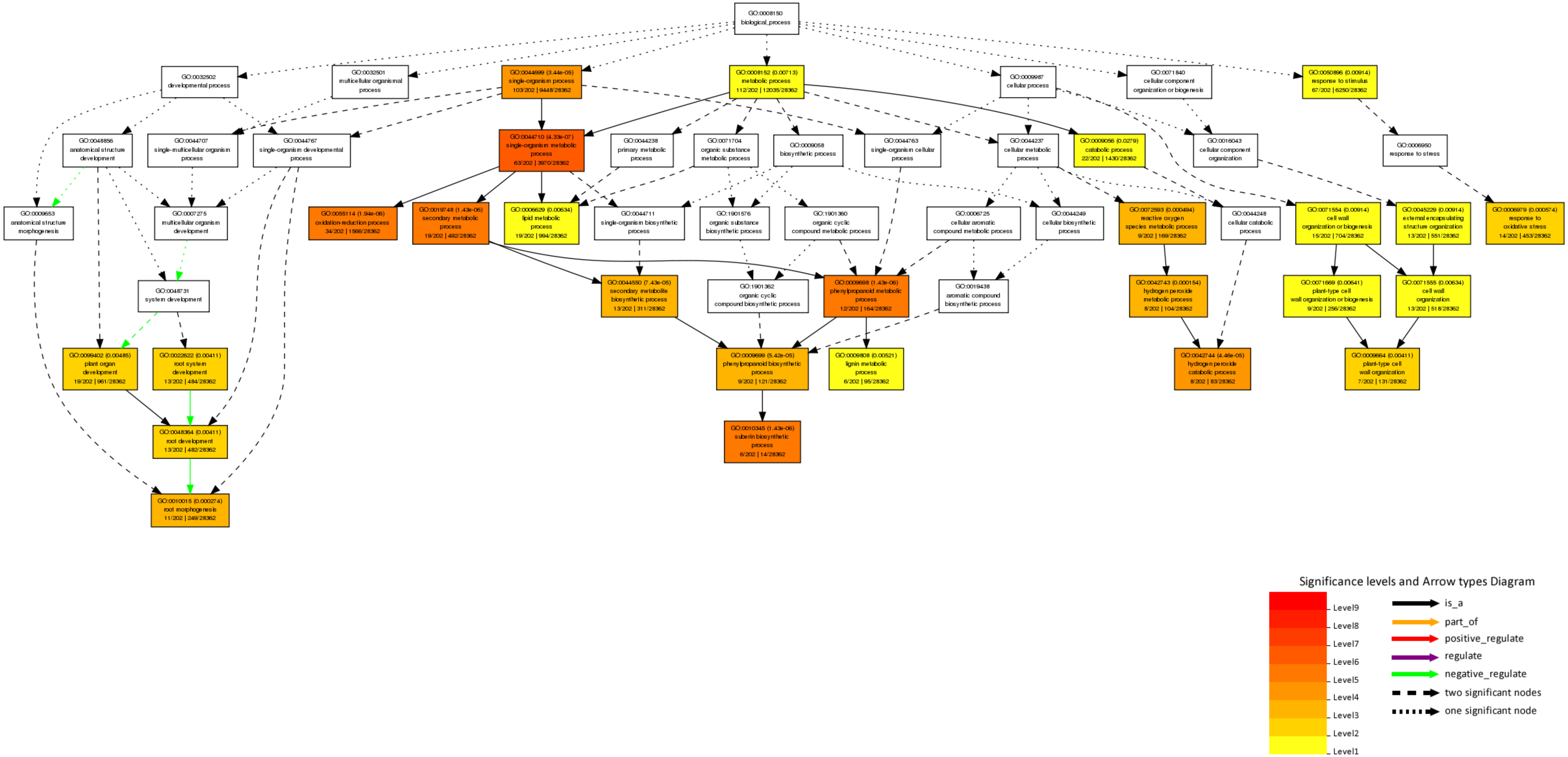

Figure S7. The enriched GO terms for upregulated genes after additional γ-irradiation of seeds from the accession Bab-0 in comparison with the reference non-irradiated seeds of the same accession (Molecular Function dictionary)

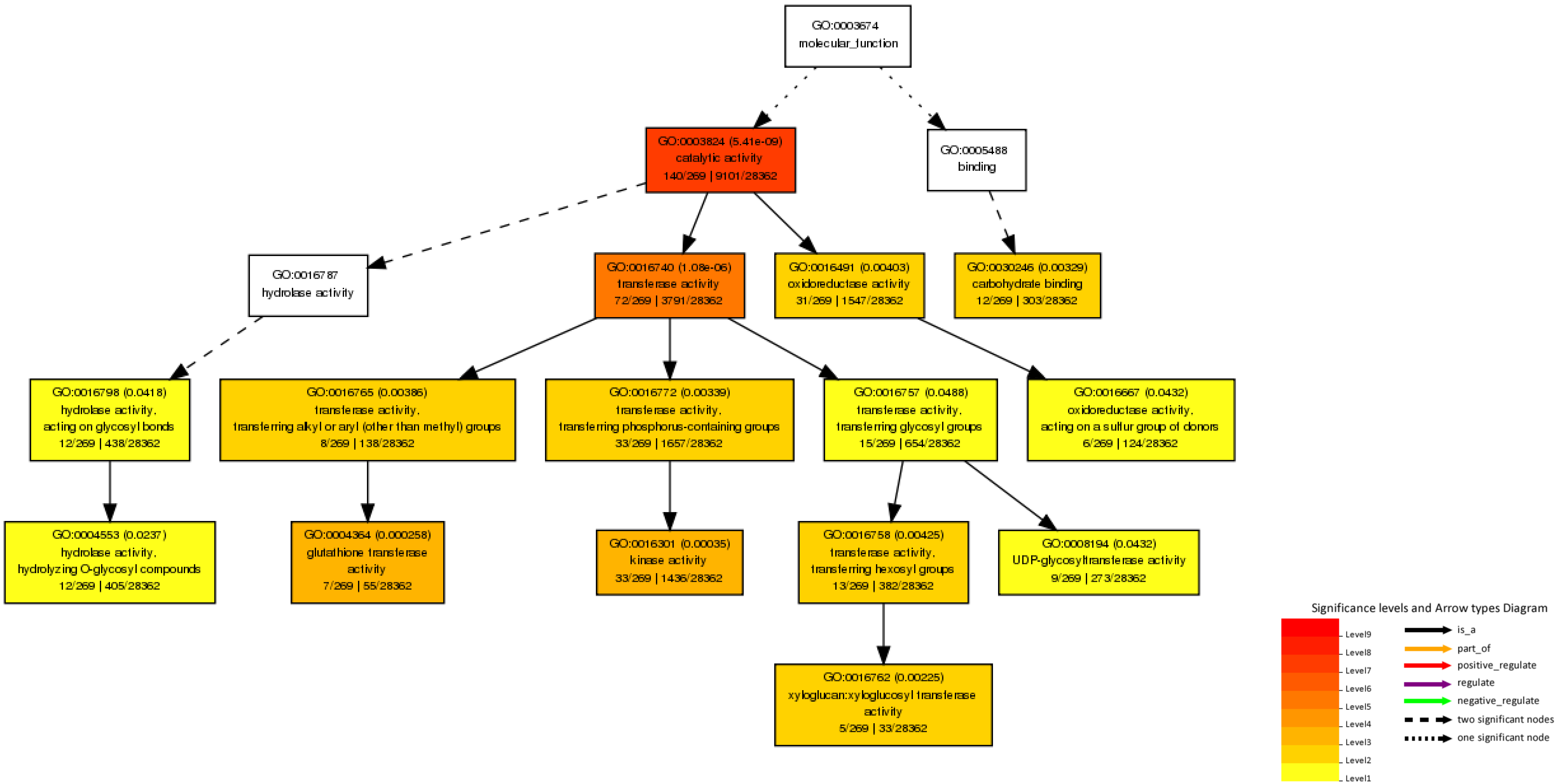

Figure S8. The enriched GO terms for downregulated genes after additional γ-irradiation of seeds from the accession Bab-0 in comparison with the reference non-irradiated seeds of the same accession (Molecular Function dictionary)

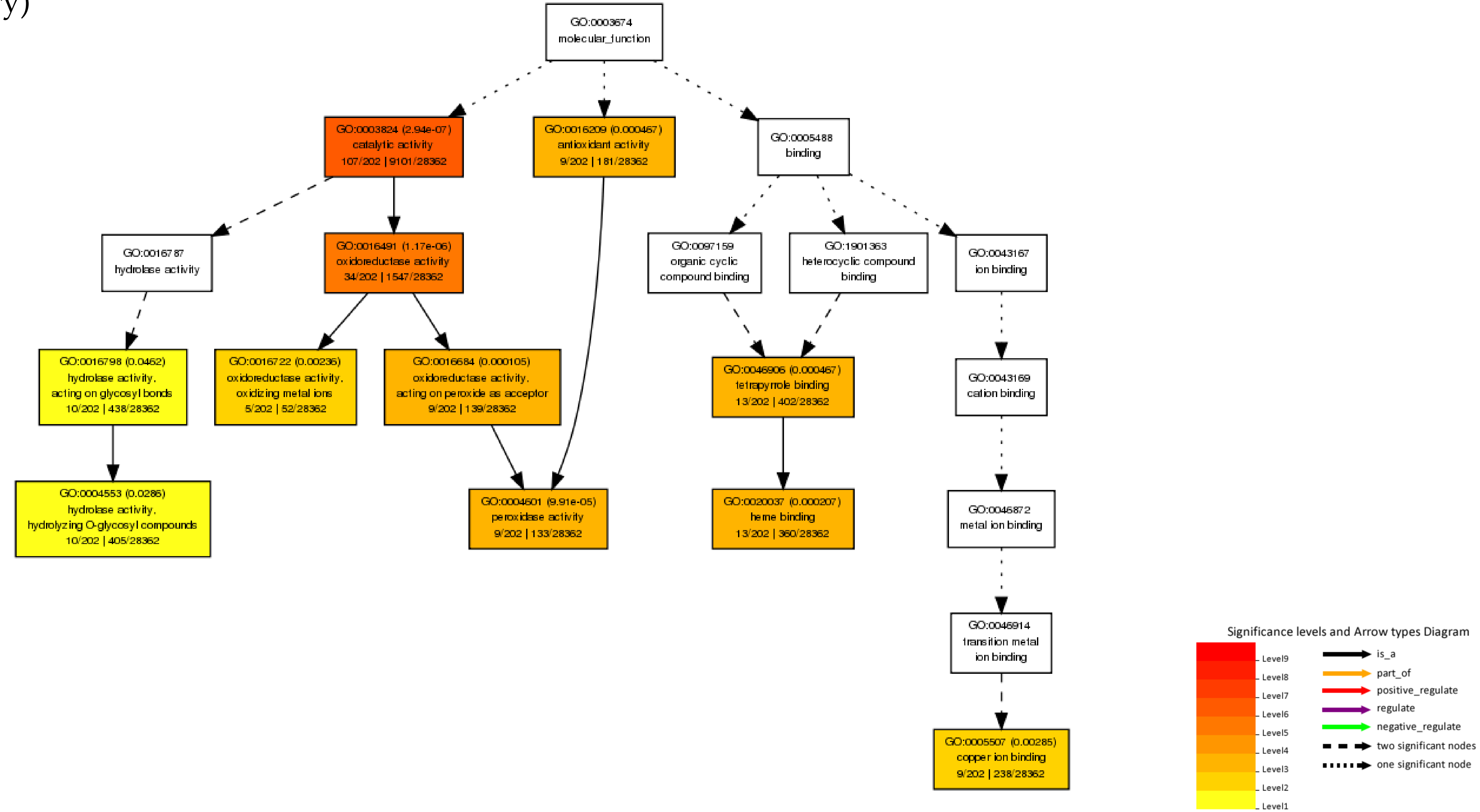

Figure S9. The enriched GO terms for upregulated genes after additional γ-irradiation of seeds from the accession Bab-0 in comparison with the reference non-irradiated seeds of the same accession (Cellular Component dictionary)

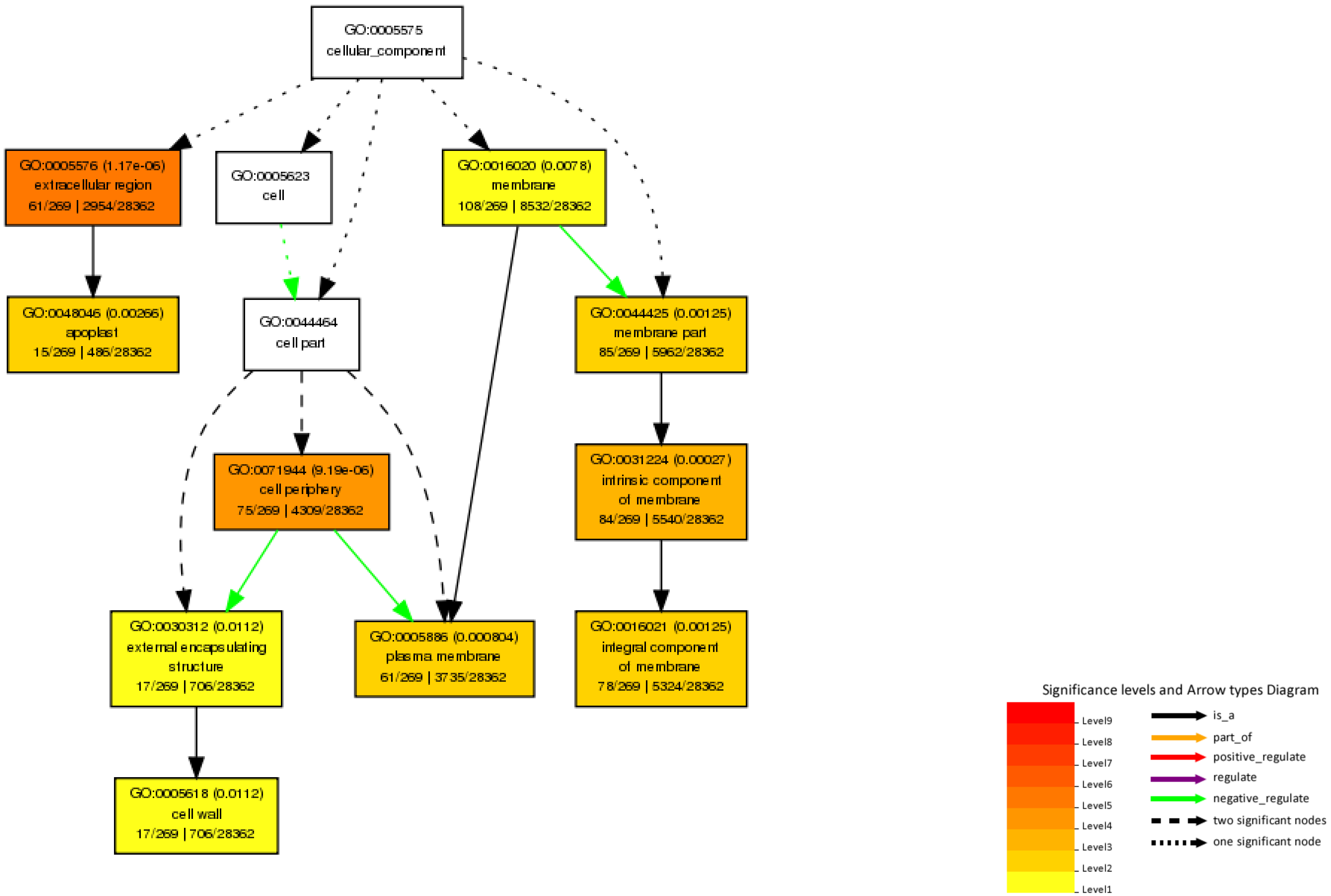

Figure S10. The enriched GO terms for downregulated genes after additional γ-irradiation of seeds from the accession Bab-0 in comparison with the reference non-irradiated seeds of the same accession (Cellular Component dictionary)

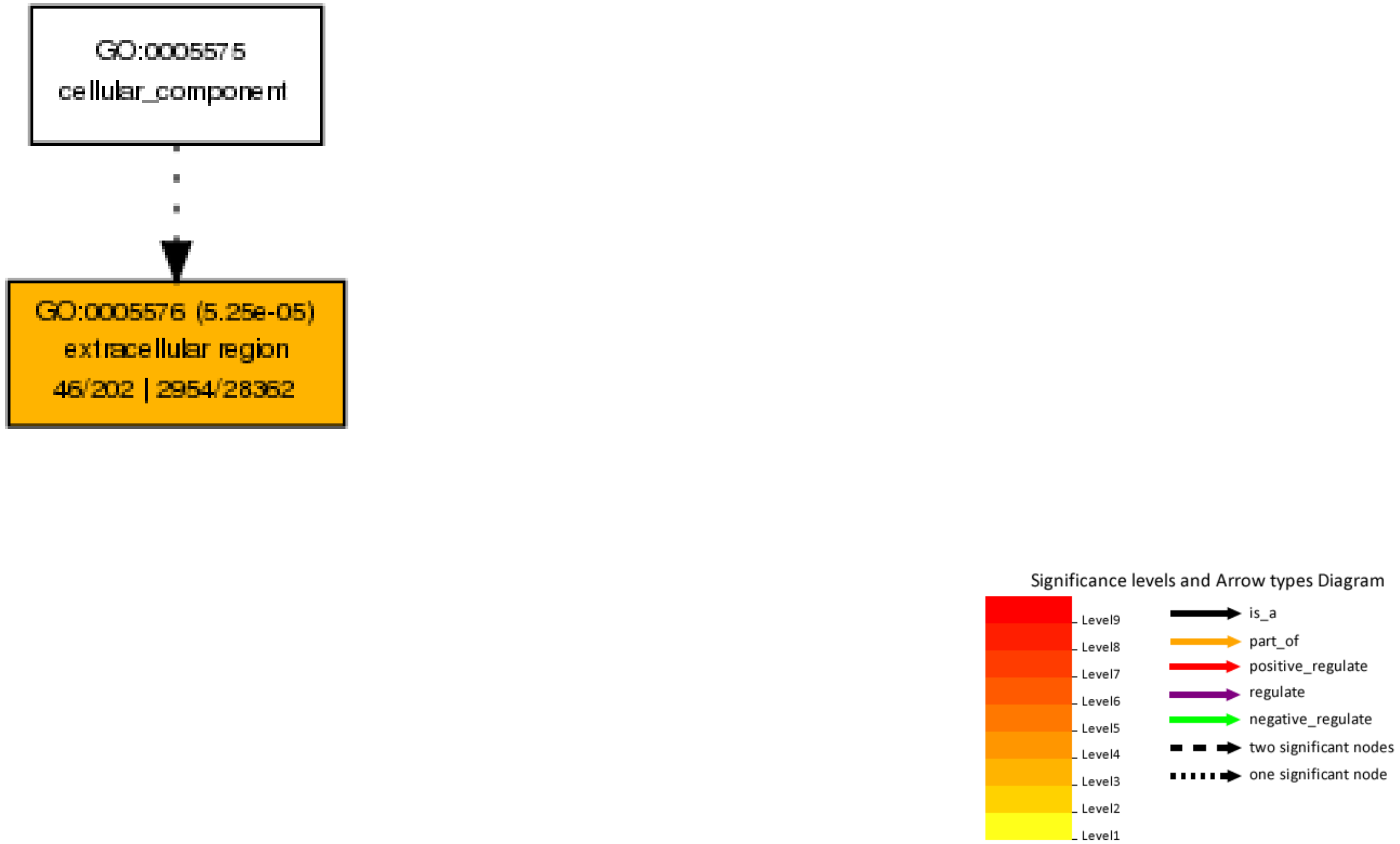

Figure S11. The enriched GO terms for upregulated genes after additional γ-irradiation of seeds from the accession VS-0 in comparison with the seeds of the same accession without acute irradiation (Biological Process dictionary)

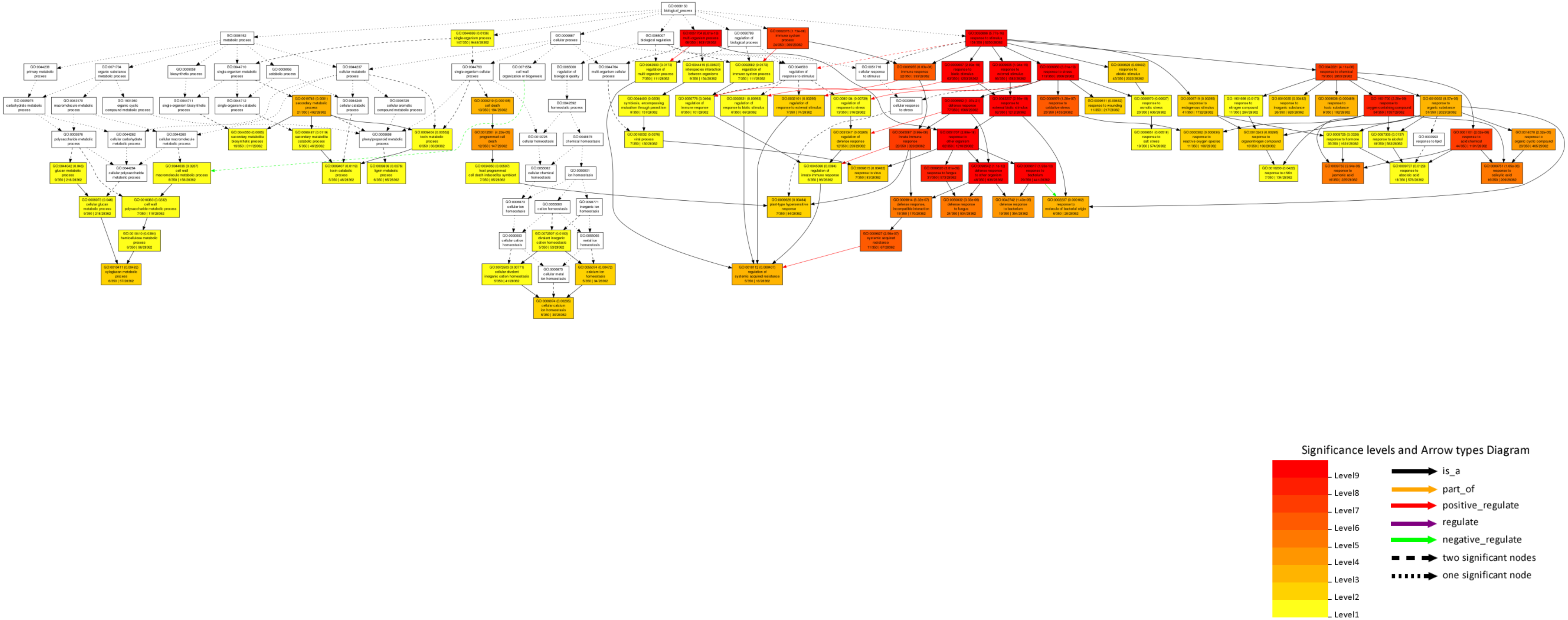

Figure S12. The enriched GO terms for downregulated genes after additional γ-irradiation of seeds from the accession VS-0 in comparison with the seeds of the same accession without acute irradiation (Biological Process dictionary)

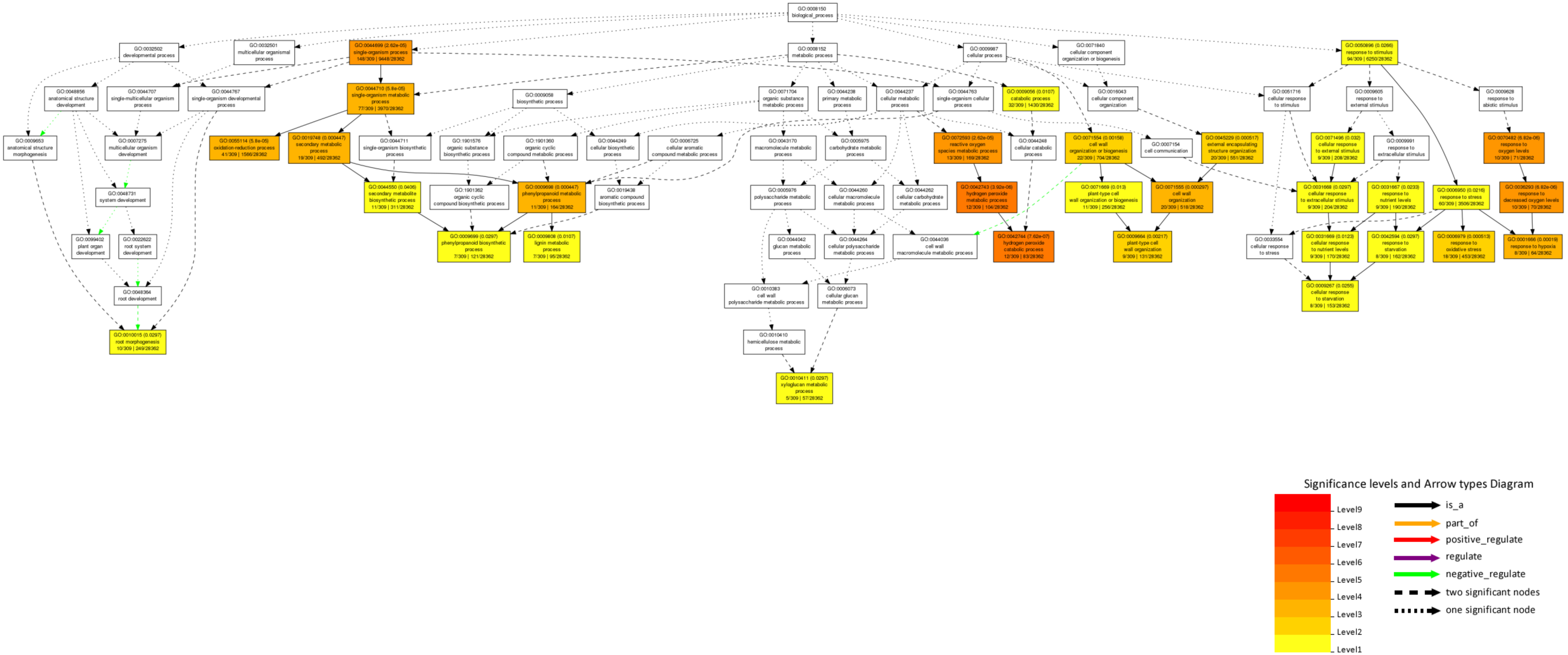

Figure S13. The enriched GO terms for upregulated genes after additional γ-irradiation of seeds from the accession VS-0 in comparison with the seeds of the same accession without acute irradiation (Molecular Function dictionary)

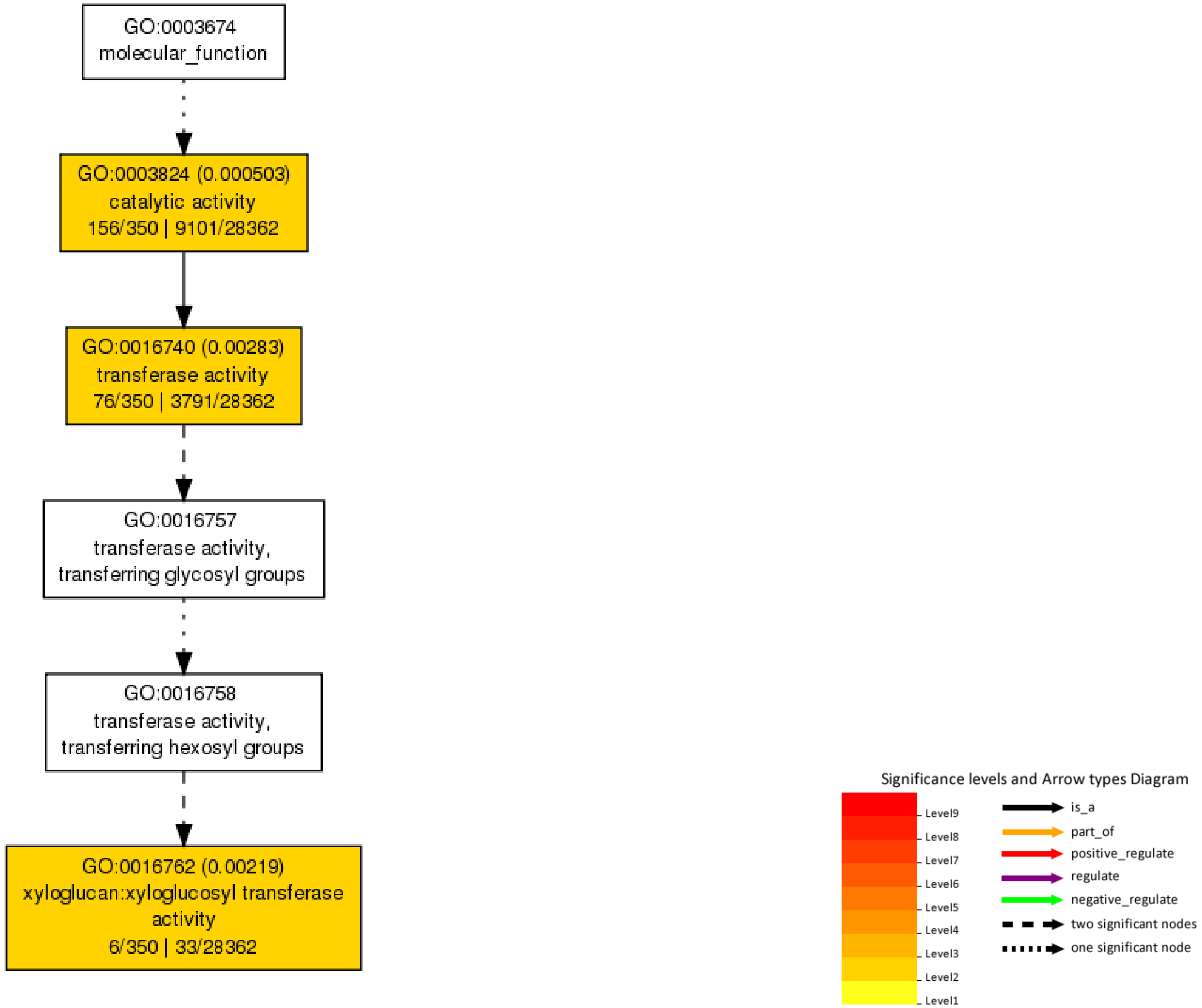

Figure S14. The enriched GO terms for downregulated genes after additional γ-irradiation of seeds from the accession VS-0 in comparison with the seeds of the same accession without acute irradiation (Molecular Function dictionary)

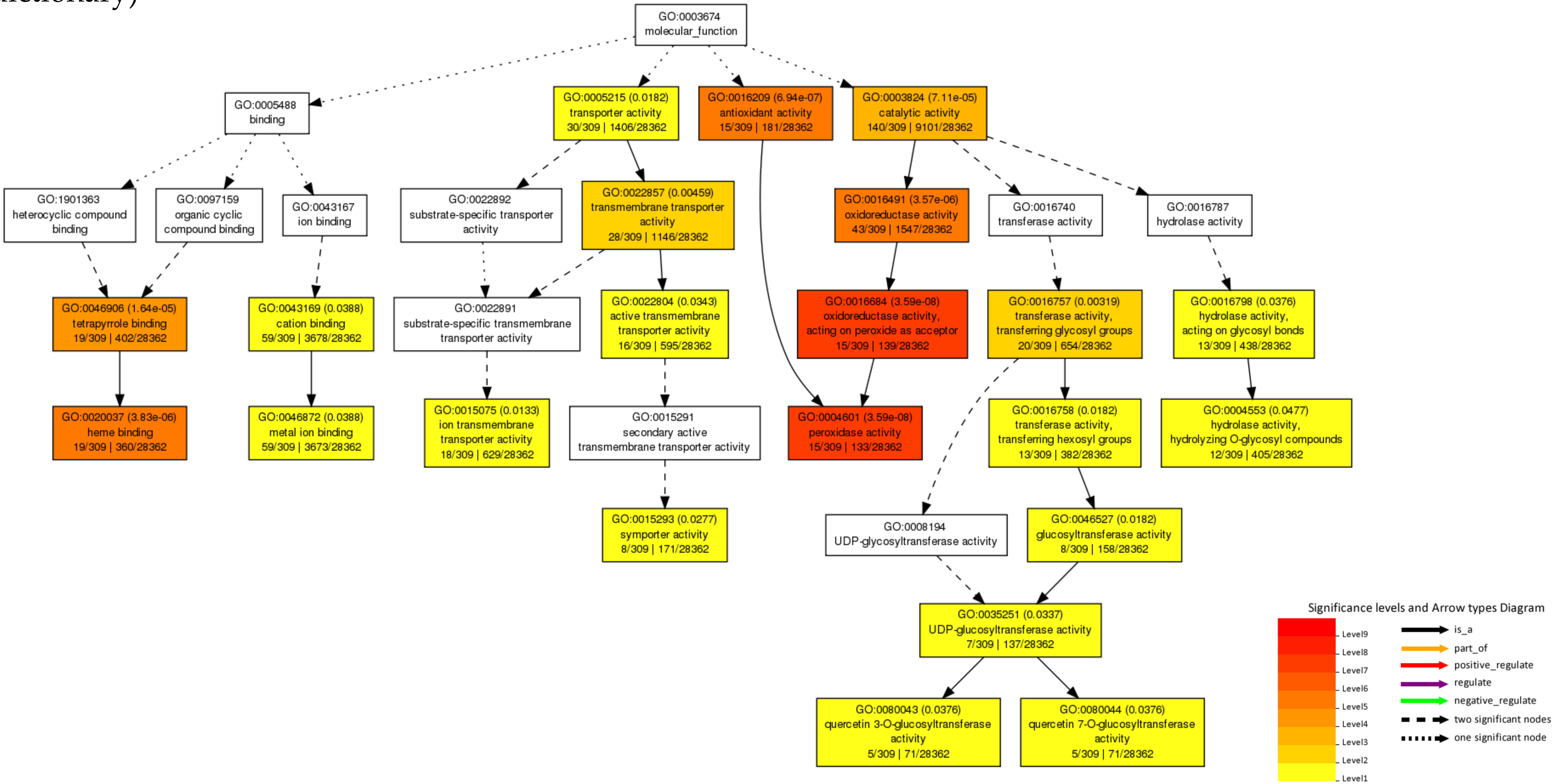

Figure S15. The enriched GO terms for upregulated genes after additional γ-irradiation of seeds from the accession VS-0 in comparison with the seeds of the same accession without acute irradiation (Cellular Component dictionary)

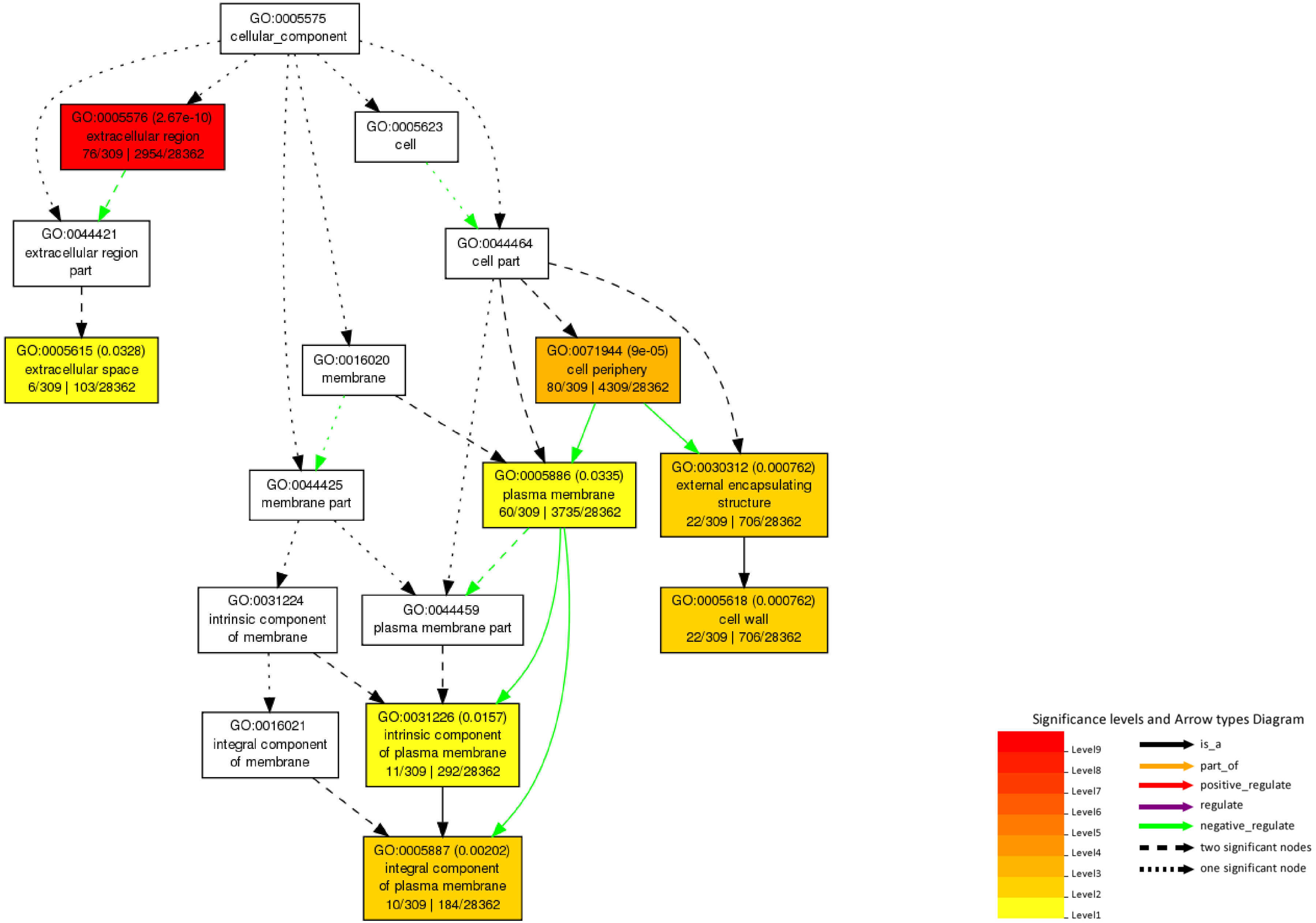

Figure S16. The enriched GO terms for downregulated genes after additional γ-irradiation of seeds from the accession VS-0 in comparison with the seeds of the same accession without acute irradiation (Cellular Component dictionary)

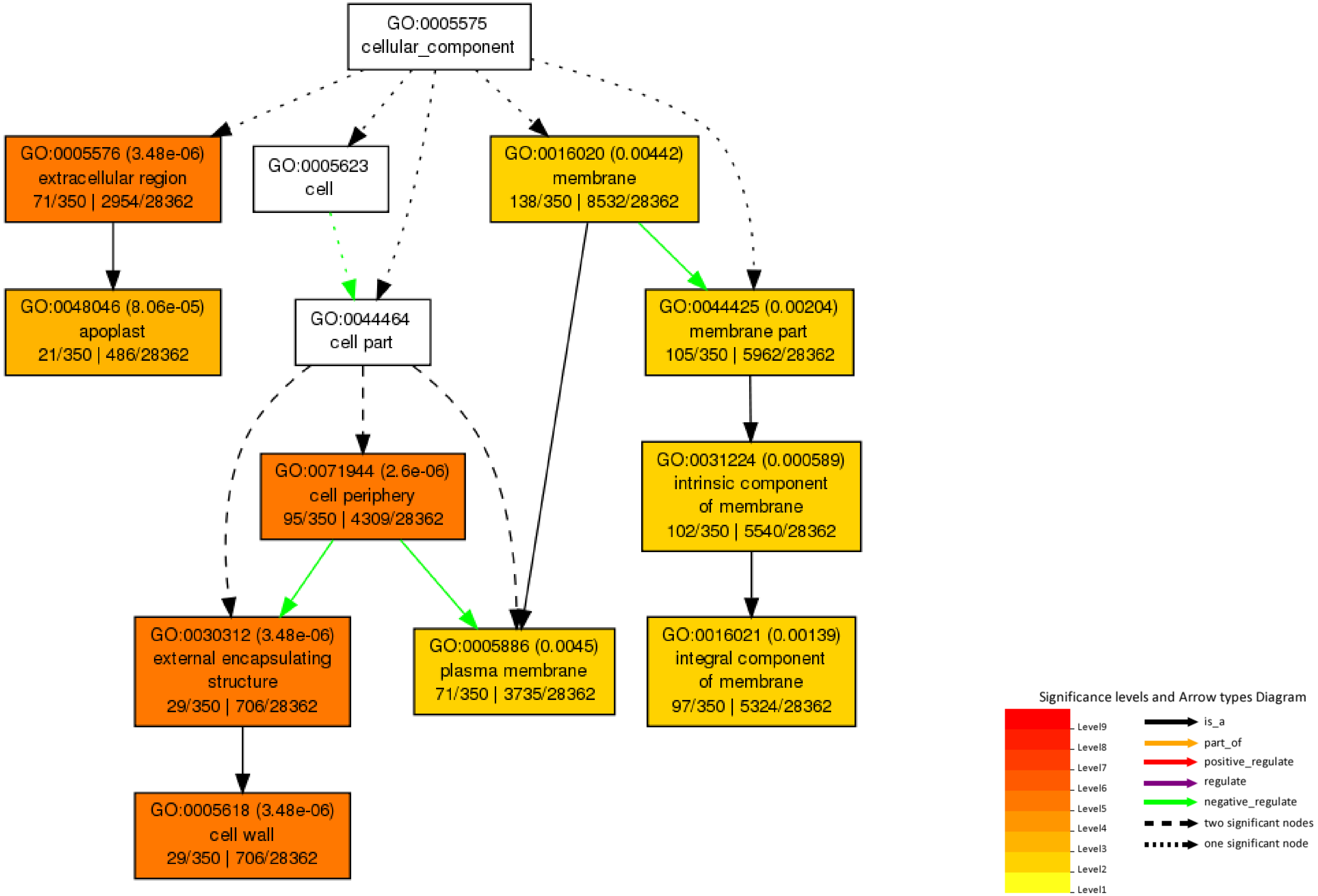

Figure S17. The enriched GO terms for upregulated genes after additional γ-irradiation of seeds from the accession Masa-0 in comparison with the seeds of the same accession without acute irradiation (Biological Process dictionary)

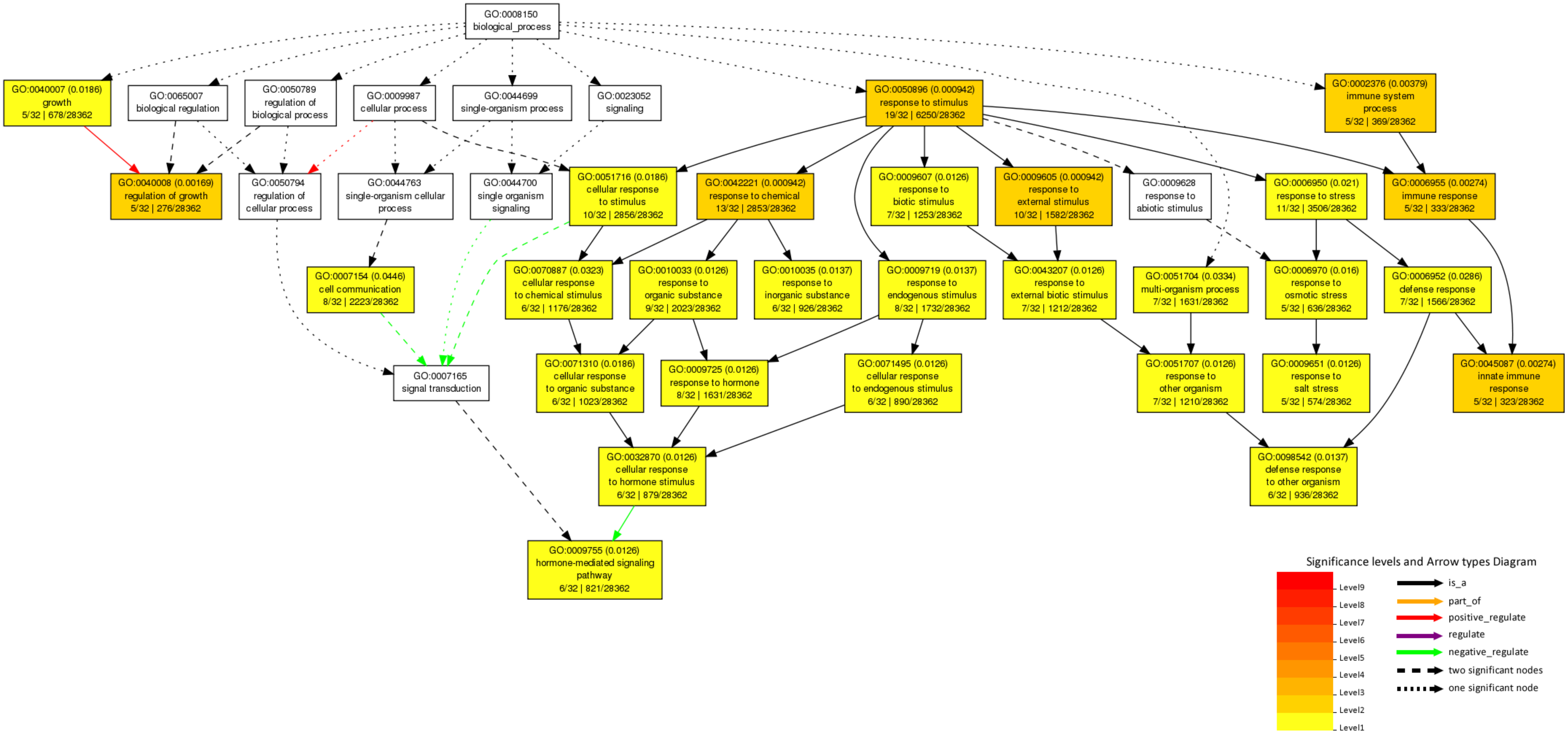

Figure S18. The enriched GO terms for upregulated genes after additional γ-irradiation of seeds from the accession Masa-0 in comparison with the seeds of the same accession without acute irradiation(Cellular Component dictionary)

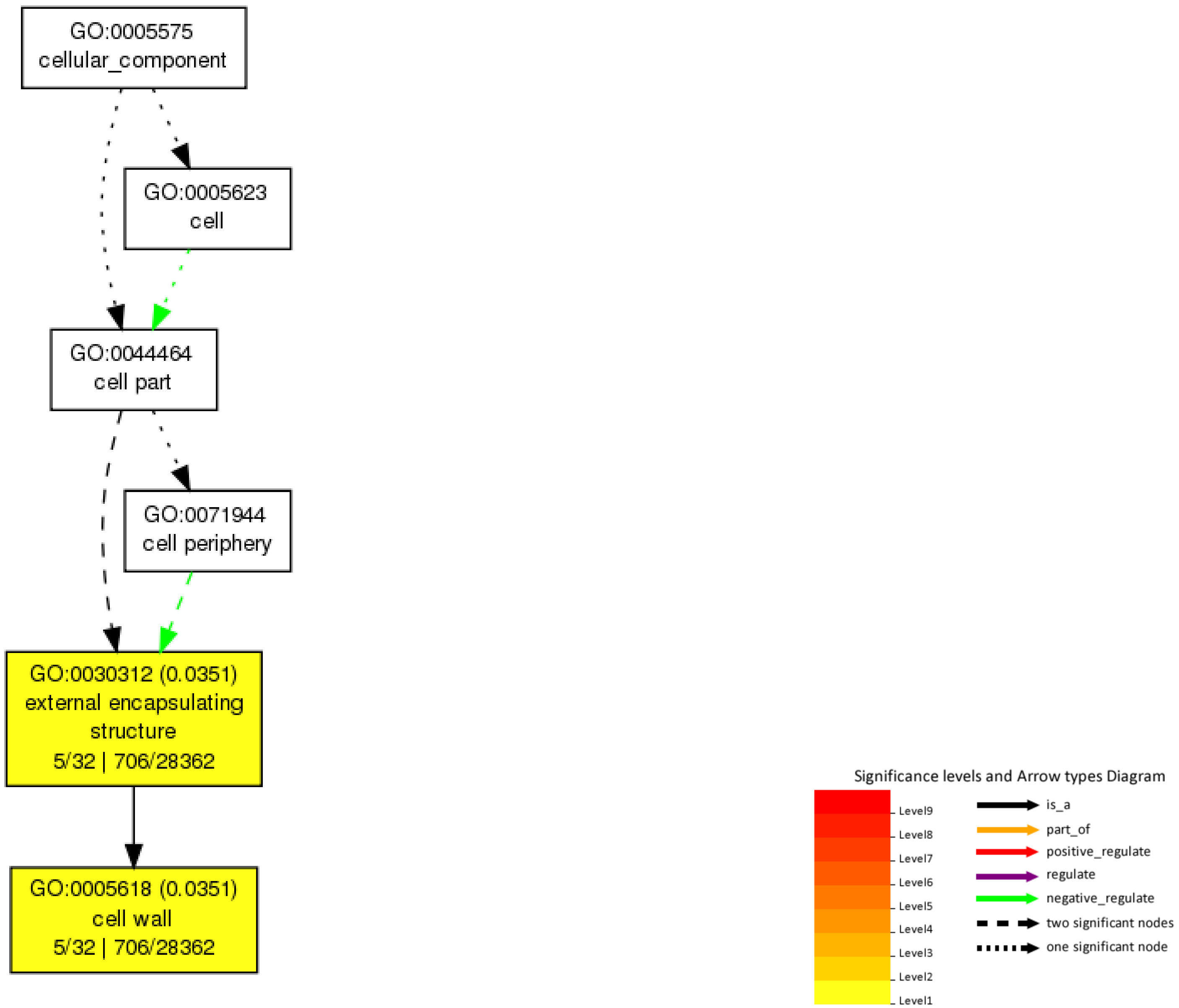

Supplement: Supplementary file 1 [file plants-11-03142-s001.zip › Figures S1-S18.pdf]
