# Supplementary material for: Arabidopsis thaliana Accessions from the Chernobyl Exclusion Zone Show Decreased Sensitivity to Additional Acute Irradiation
Source: Plants (Basel). 2022 Nov 17;11(22):3142. doi: 10.3390/plants11223142 (PMC9697804; doi:10.3390/plants11223142)

Figure S19. Photos of the experimental plot Babchin (Polesye State Radiation-Ecological Reservee, Gomel Region, Republic of Belarus)
GPS coordinates : 51.791470, 30.007070

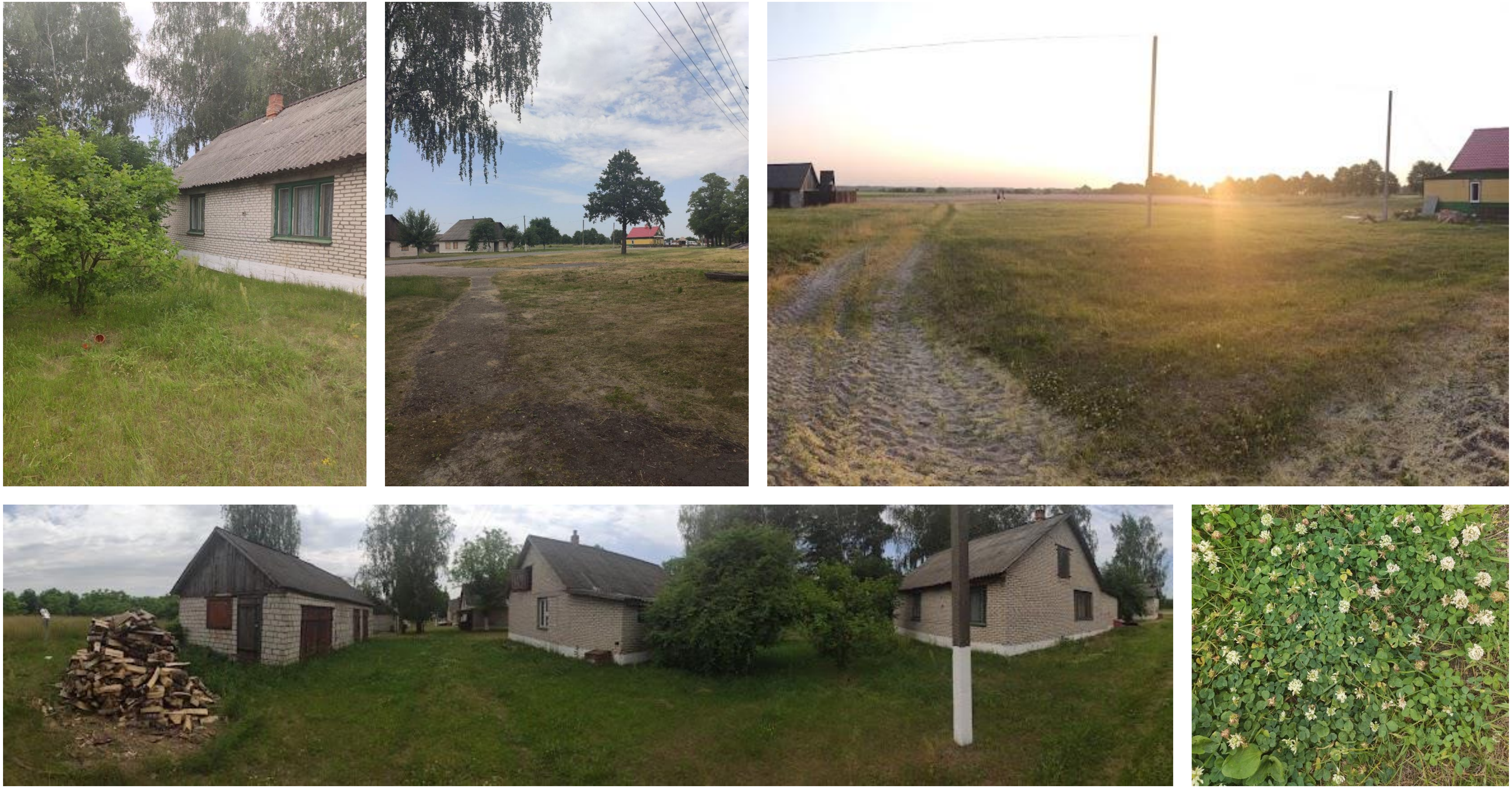

Figure S20. Photos of the experimental plot Vygrebnaya Sloboda (Polesye State Radiation-Ecological Reserve, Gomel Region, Republic of Belarus)
GPS coordinates: 51.650500, 30.135470

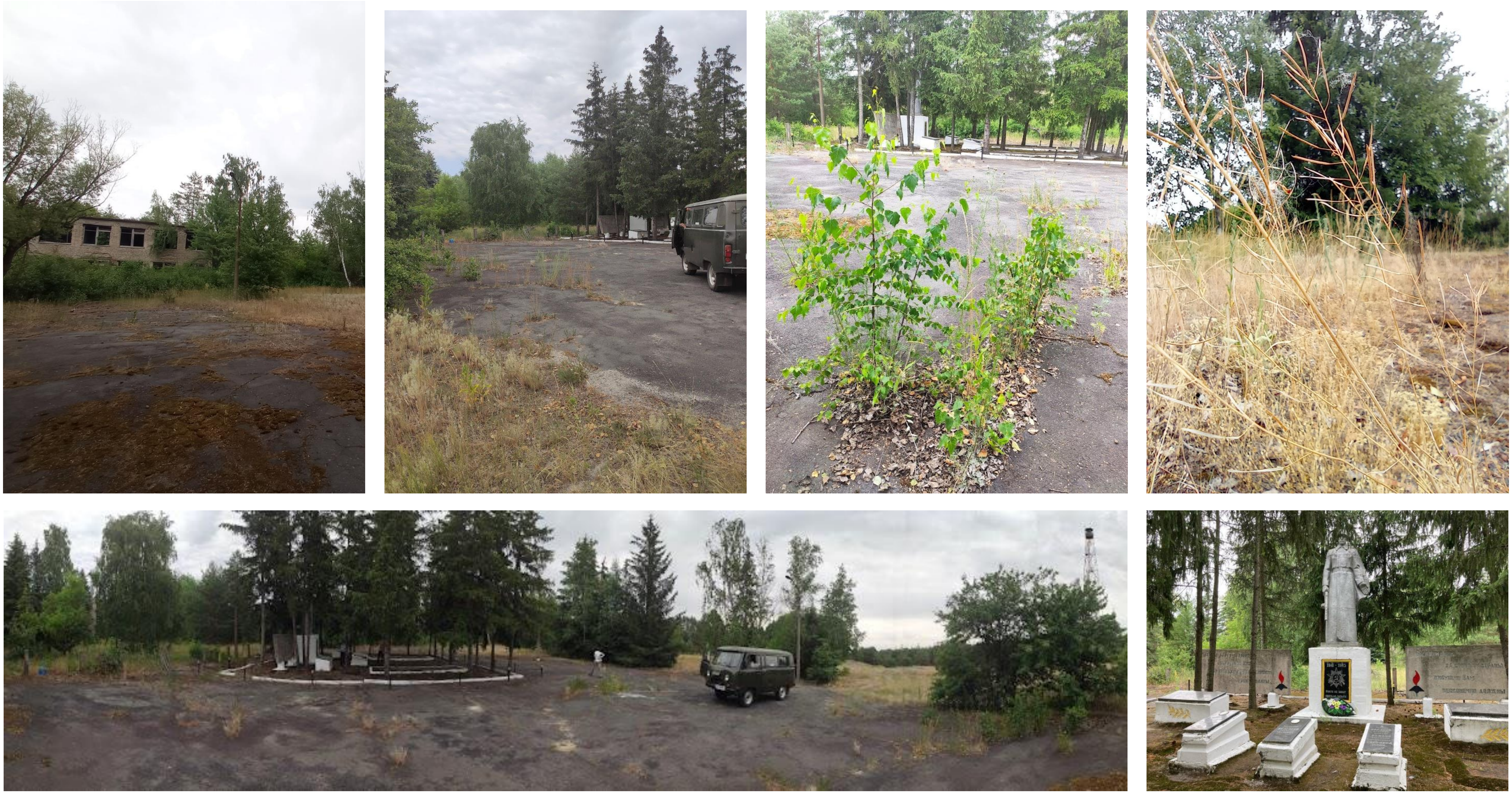

Figure S21. Photos of the experimental plot Masany (Polesye State Radiation-Ecological Reserve, Gomel Region, Republic of Belarus)
GPS coordinates: 51.513210, 30.020740

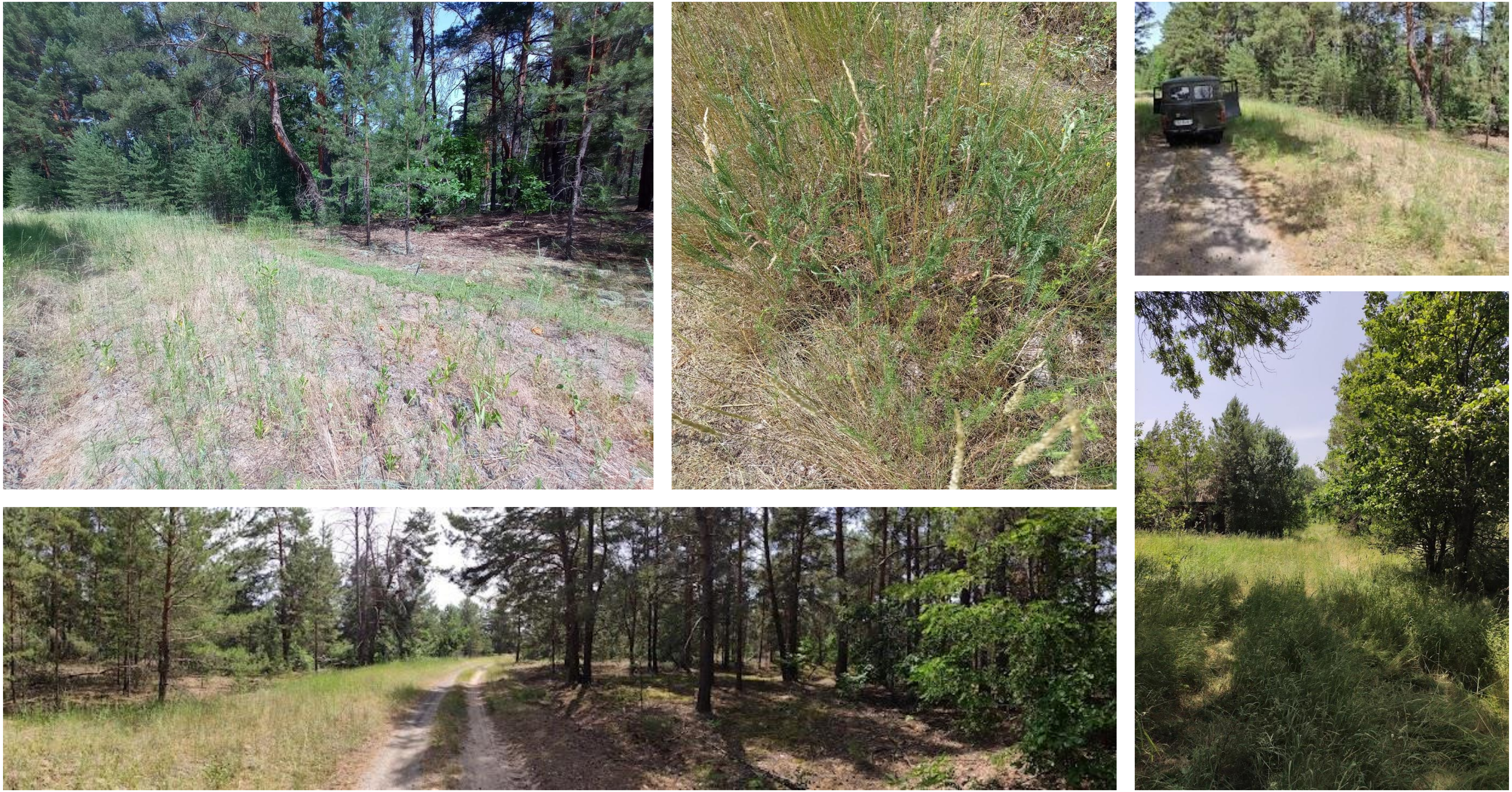

Supplement: Supplementary file 1 [file plants-11-03142-s001.zip › Figures S19-S21.pdf]
